# Pathogenic potential of polyextremotolerant fungi in a warming world

**DOI:** 10.1371/journal.ppat.1013102

**Published:** 2025-04-30

**Authors:** Erin C. Carr, Erin L. Bredeweg, Grace E. Hamilton, Tania Kurbessoian, Audrey M. Williams

**Affiliations:** 1 Department of Chemical and Biomolecular Engineering, University of Nebraska-Lincoln, Lincoln, Nebraska, United States of America; 2 Environmental and Molecular Sciences Division, Pacific Northwest National Laboratory, Richland, Washington, United States of America; 3 Department of Chemistry, High Point University, High Point, North Carolina, United States of America; 4 Department of Microbiology and Plant Pathology, Institute for Integrative Genome Biology, University of California, Riverside, California, United States of America; 5 Department of Cell Biology, Duke University, Durham, North Carolina, United States of America; University of Maryland, Baltimore, UNITED STATES OF AMERICA

## Introduction

This review focuses on opportunistic human pathogens found in the broad class of polyextremotolerant fungi (PEF) and how climate change will affect these organisms and their pathogenic potential.

## What are polyextremotolerant fungi (PEF)?

**Fig 1.**
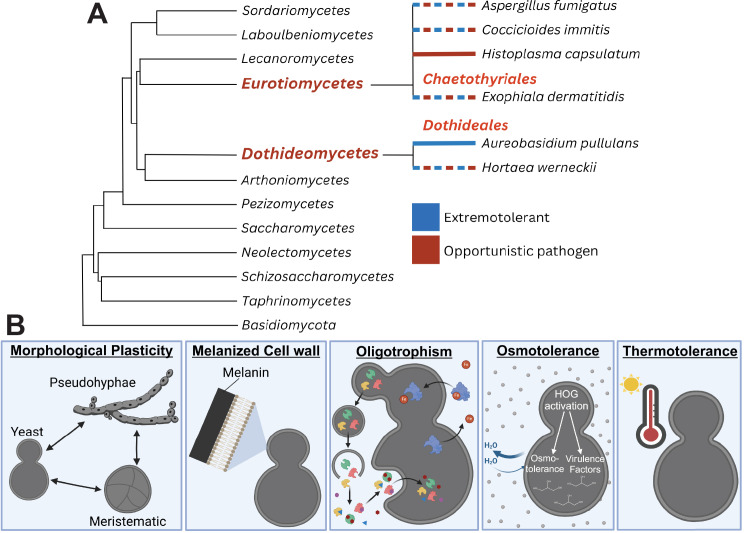
(A) Adapted from Gostinčar and colleagues [3] used under CC BY 4.0, this dendrogram depicts *Ascomycetes* within the orders *Eurotiomycetes* and *Dothiomycetes* that include the polyextremotolerant fungi classes *Chaetothyriales* and *Dothideales*. The evolutionary development of multi-stressor tolerance is associated with multiple fungal lifestyles (colored lines). The blue lineage lines indicate extremotolerant species, the red lines indicate opportunistic pathogens, and mixed-dashed lines indicate observation of both roles. (B) This figure illustrates the variety of cell traits polyextremotolerant fungi exhibit that contribute to both extremotolerance and pathogenicity. PEF cells have features like melanization and morphological plasticity, providing the cell with physical advantages. Molecular features, including siderophore expression, thermotolerance, and the presence of a modified HOG pathway, enable these species to grow in extreme environments (not to scale).

The fungal kingdom includes some of the planet’s hardiest organisms, capable of surviving physiochemical extremes of temperature, low water availability, UV exposure, and nutrient limitation far outside the survivable range of most organisms. PEF are capable of growing in multiple of these extremes [1]. Members of this group are found primarily in two classes: *Dothideomycetes* (*Dothideales*) and *Eurotiomycetes* (*Chaetothyriales*) (Fig 1A). In addition to their growth under extreme conditions, members of this polyphyletic group are united by several frequently observed cellular traits including melanized cell walls, slow growth rates, and high degrees of morphological plasticity [2]. These traits—and almost certainly others we have yet to discover—are thought to allow these fungi to grow in high alpine habitats, antarctic deserts, nuclear reactor meltdown sites, and other harsh environments colonized by few other organisms.

Many PEF are cosmopolitan and widespread in the human-built anthroposphere. Although some species’ anthroposphere colonization may be incidental to their extremotolerance, adaptations that allow fungi to grow on barren rock surfaces likely also facilitate their growth on built surfaces, and adaptations that enable fungi to grow in extreme heat may similarly enable them to weather our dishwasher cycles [3]. Among fungi, PEF are overrepresented as human opportunistic pathogens, suggesting that the same adaptations that let these fungi survive physiochemical extremes also facilitate survival and growth in humans [3]. Many fundamental questions remain about this group, including which adaptations allow them to grow in extremes, the links between extremotolerance and pathogenesis, and whether knowledge of those adaptations can help us treat human disease or anticipate the emergence of new pathogens.

## What is the connection between polyextremotolerance and pathogenesis?

Physiological features of PEF that enable their polyextremotolerance have allowed them to adapt to a wide range of lifestyles. Therefore, PEF can become opportunistic human pathogens, as their extensive toolbox of characteristics has allowed for dissemination in the body. Opportunistic pathogens among PEF exhibit traits that are associated with pathogens of endotherms: the ability to grow at 37 °C, osmotic resistance, oligotrophism, morphological plasticity, and melanization [3] (Fig 1B).

Perhaps the most important trait for human pathogens is the ability to grow at 37 °C, i.e., thermotolerance [4]. This, however, is a rare trait—not just amongst PEF, but throughout the fungal kingdom [4]. This is also the only trait that has (thus far) been found in *all* pathogenic fungi [3]. Some PEF also have high osmotic resistance due to a modified high osmolarity glycerol (HOG) pathway [5] (Fig 1B). The HOG pathway, initially discovered for its ability to regulate osmolarity, is also linked to the activation of the two-component system that regulates virulence factors [5]. PEF with an enhanced HOG pathway may also express virulence factors, triggering opportunistic pathogenesis, but this has yet to be determined [2].

PEF are well known for their ability to grow in oligotrophic ecosystems. This feature is crucial for surviving in the human body due to the challenges of nutrient acquisition. Traits of increased extracellular enzyme and siderophore production [6] simplify nutrient access in the human body. Finally, a striking feature of PEF is their melanin. While pigmentation is not required for pathogens, it seems to be necessary for pathogenesis by those fungi that produce it [7]. Specifically, deletion of the first gene in the melanin production pathway reduces virulence in both PEF, such as *Exophiala dermatitidis*, and others, such as *Cryptococcus neoformans* [7]. This is likely because melanin’s properties provide multiple abiotic protections against ROS, metals, antifungal drugs, and phagocytic cells, which assists in defending against the human immune system [7].

## What human diseases do PEF cause?

PEF can cause disease in immunocompromised humans and—more rarely—immunocompetent ones. Well-known disease-causing fungal species described in the World Health Organization watch list include *Aspergillus* spp*.*, *Histoplasma capsulatum*, *Candida* spp*.*, *Coccidiodes* spp*.*, and *C. neoformans* [8]. More than 100 species of PEF can cause phaeohyphomycosis and chromoblastomycosis. Phaeohyphomycosis is a type of infection due to the presence of highly melanized fungi found in different parts of the human body, including superficial nodules under the skin, abscesses, cysts, and lesions [9]. Disseminated cases can be found in the eyes, bones, heart, and brain. Chromoblastomycosis is differentiated by subcutaneous cauliflower-like lesions that can ulcerate [9]. PEF that cause disease include *Exophiala* spp*.* (Fig 1A), *Alternaria* spp*.*, *Rhinocladiella mackenzie*, *Cladophialophora* spp., *Phialophora* spp., and *Fonsecaea* spp. Other well-known disease states of PEF include opportunistic infections of individuals with cystic fibrosis. Fungal species involved with cystic fibrosis secondary infections include *Scedosporium apiospermum*, *Aspergillus fumigatus*, *Candida albicans*, *Clavispora (Candida) lusitaniae*, and PEF *E. dermatitidis* [10].

There was an increase in human phaeohyphomycosis and chromoblastomycosis cases in the recent century [9]. This is likely due to an increase in built environments, including dishwashers, which can harbor PEF like *Exophiala* spp*.* [3]*.* It has also been postulated that *E. dermatitidis*, an opportunistic human pathogen, has broad natural reservoirs including wasp nests, healthy bats, lesions on toads, and rotten wood [11]. This suggests human patients can be infected through environmental exposure, which could be the case for many PEF infections still not described [11].

A specific fungal virulence factor seen in known human pathogens like *H. capsulatum* and *C. albicans* is a dimorphic switch in the human body due to temperature increase and other factors [12]. This echoes highly dimorphic (meristematic) features of PEF (Fig 1B), which are very specific to this group and may be one of the ways PEF pose a pathogenic threat to human populations.

## How is global warming exacerbating the threat?

Mammals retain high and constant body temperatures relative to ectothermic organisms that suffer much higher burdens of fungal disease; it has even been proposed that mammalian endothermy evolved, at least in part, as an adaptation conferring resistance to fungal pathogens [13]. Thermotolerance is thus necessary (but insufficient) for fungal pathogenesis in humans [4]. Global warming, by raising average temperatures worldwide and increasing the incidence of extreme temperature events, likely exerts selective pressure for increased thermotolerance on all of the estimated 5 million species of the fungal kingdom.

Zoonosis is a major source of human infectious diseases, such as SARS-CoV2. Fungal pathogens of ectothermic animals already possess molecular traits necessary for virulence in animal hosts. If such fungal pathogens became thermotolerant, they would likely pose an increased zoonotic threat to humans. Newly thermotolerant environmental fungi also represent a potential source of novel human pathogens. Casadevall and colleagues argue that *Candida auris*—a plant saprophyte that independently emerged as a human pathogen in Africa, Asia, and South America in the past 15 years—may represent “the first example of new pathogenic fungi emerging from climate change” [14].

PEF have a head start adapting to a warming planet. Although most fungi are mesophilic, with growth optima of 25–35 °C [8,15], thermotolerance is a feature of many PEF. Within the polyextremotolerant genus *Aureobasidium*, both increased thermotolerance and increased melanization correlated with greater virulence in a murine infection model [16]. Even among those who question the premise that increased fungal thermotolerance will drive the emergence of *new* fungal pathogens, there is a consensus that climate change is already expanding the range of *existing* fungal pathogens [17].

Extremotolerant fungi also show promise in protecting plants from heat and drought and acting as biocontrol agents, making them potentially valuable agricultural tools as the earth’s farmland warms [18–20]. However, it will be important for us to utilize species with traits that increase crop resilience while lacking traits that make them potential opportunistic pathogens. The relative threat of new and existing pathogens would be elucidated by a deeper understanding of how fungi adapt to novel, hostile, and changeable environments, i.e., fungal extremotolerance.

## What can we as scientists do to address the problem?

Fungal pathogens cause over 150 million infections and 1.7 million deaths annually [21]. Though we have identified PEF and pathogenic traits like thermotolerance, melanin production, and morphological plasticity [3] (Fig 1B), which can change fungal antigenic structures during immune evasion [16], we are still missing aspects of the host-pathogen interaction, symptom identification, and interventions. The evolutionary diversity of PEF represents a challenge for identifying gene functions using bioinformatics, and significant proportions of fungal genes remain uncharacterized.

As scientists engaged in discovering new taxa and analyzing the genetic and phenotypic characteristics of PEF, we are well-placed to develop identification and diagnostic tools. The interface of fungal and human biology is an opportunity for studies of extremotolerance mechanisms, antifungal development, and resources to share knowledge between fungal biologists and the medical community.

A nuanced understanding of PEF cell biology is critical for the effective diagnosis, treatment, and prevention of emerging mycoses. To address this, the PEF community should expand and coordinate functional annotation of fungal genomes, in an effort to identify common drivers of both pathogenesis and extremotolerance, including cryptic and uniquely fungal genes. Those familiar with the unique biology of PEF can aid in the development of new treatments for these opportunistic pathogens, such as novel antifungal compounds and delivery methods [22], and vaccine development using live attenuated or killed cell material, including recognition of cell-surface polysaccharides or proteins, or recombinant protein antigens unique to fungi [23]. While there are currently no FDA-approved vaccines to protect humans against fungi, preliminary studies suggest that an intimate knowledge of fungal biology will allow us to not only treat but *prevent* fungal disease [24,25]. However, these efforts require identification of the molecular traits that distinguish PEF from human host cells—an effort that will be supported by powerful new tools that increase the sensitivity and range of our observations such as metabolomics, proteomics, and detection of volatile compound production. PEF play critical roles in their local environmental niches along with detrimental effects in human bodies; more effort and funding need to be allocated to curb their pathogenic potential in our warming world.
